# The Influence of Barley Proteome on Hop Bitter Acid Yield during Brewing

**DOI:** 10.1021/acs.jafc.4c04396

**Published:** 2024-09-16

**Authors:** Mariana B. C. Pinto, Flavio L. Schmidt, Zhuo Chen, Juri Rappsilber, Brian Gibson, Philip C. Wietstock

**Affiliations:** † Fruit and Vegetables Laboratory−Department of Food Technology, School of Food Engineering, University of Campinas (UNICAMP), R. Monteiro Lobato 80, 13083-862 Campinas, São Paulo Brazil; ‡ Institut of Food Technology and Food Chemistry, Chair of Brewing and Beverage Technology, Technische Universität Berlin, 13353 Berlin, Germany; § Bioanalytics, Institute of Biotechnology, Technische Universität Berlin, 10623 Berlin, Germany

**Keywords:** *Humulus lupulus*, proteomics, isohumulones, circular economy, process efficiency

## Abstract

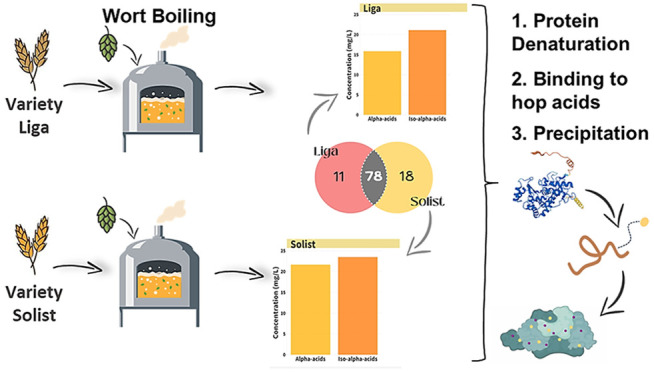

A persistent challenge in brewing is the efficient utilization of hop bitter acids, with about 50% of these compounds precipitating with trub during wort boiling. This study aims to uncover the correlation between the barley cultivar proteome and hop bitter acid utilization during wort boiling. Therefore, comparative experiments were conducted using two cultivars, Liga and Solist, with varying proteomes to identify specific proteins’ role in hop bitter acids precipitation. High-performance liquid chromatography (HPLC) was used to measure hop bitter acid content, while liquid chromatography-tandem mass spectrometry (LC-MS/MS) was used to quantify and identify proteins. The 107 protein groups, particularly enzymes linked to barley metabolic defense mechanisms, exhibited significant differences between the two cultivars. Results revealed significantly lower α- and *iso*-α-acid content in wort produced from the barley cultivar Liga. This study highlights the critical role of the barley proteome in optimizing process efficiency by enhancing hop utilization through barley cultivar selection.

## Introduction

The global brewing industry holds a significant position as a primary producer of alcoholic beverages, yielding an impressive 1.89 billion hectoliters in 2022.^1^ However, this achievement comes with a notable environmental cost, marked by a substantial greenhouse gas (GHG) emission of approximately 30,172.15 kilotons of CO_2_ equivalent, with 22% originating from the supply chain, particularly raw materials.^2^ Moreover, the industry faces the generation of considerable quantities of by-products, amounting to 4352 kilotons of solid waste in 2021.^3^ In light of the pressing global challenge of climate change, the brewing industry faces a pivotal moment, inducing a change from a linear business model to one rooted in the principles of a circular economy. The circular economy, characterized by maximizing resource utilization and minimizing waste generation, serves as a strategic framework for reusing, reducing, and recycling materials, energy, and resources to foster sustainability and mitigate environmental impacts.^4,5^

In the brewing industry, optimizing the extraction of bitter compounds from hops (*Humulus lupulus L*.) presents a significant challenge, as these compounds are crucial for contributing the desired bitterness to the final product. Studies indicate that only a modest portion, typically between 30% and 50%, of the bitter acids are retained in the finished beer.^6^ This inefficiency establishes a considerable obstacle to optimizing beer production, especially for highly hopped beverages. The essential bitter compounds in beer are the *iso-α*-acids, generated by the precursor α-acids during wort boiling.^7^ However, the formation of *iso-α*-acids is reduced by factors such as protein coagulation and precipitation with trub during the boiling process. Elevated temperatures during boiling induce protein denaturation, facilitating the formation of protein–protein complexes.^8,9^ Yet, the precise interaction between hop bitter compounds and barley-derived proteins remains poorly understood.

Barley malt serves as the primary source of proteinaceous material in both wort and beer. However, a significant portion of these proteins coagulate as trub, constituting approximately 50–60% of the protein content in this by-product.^10^ The composition of the barley proteome is inherently influenced by genotype, with slight modifications occurring during the malting process.^11^ In a recent study, we explored the impact of hop bitter acids addition on the formation of protein–protein complexes during boiling, revealing alterations in protein aggregation mechanisms and protein profiles of resulting aggregates.^12^ While existing research has predominantly focused on the influence of barley genotype and proteome on beer quality traits like foam stability and haze, little attention has been paid to their role in protein precipitation during boiling and subsequent hop bitter acid utilization.^13^ Hence, this study represents a pioneering effort to elucidate the influence of barley genotype on protein coagulation during boiling and, consequently, its effect on the hop bitter acid yield. Unraveling these complex interactions will enable the brewing industry to use hop products and barley malt more efficiently, thereby promoting sustainability goals such as GHG mitigation, aligning with the United Nations’ sustainable development agenda.^14^

## Materials and Methods

### Experimental Design and Wort Preparation

**Figure 1:**
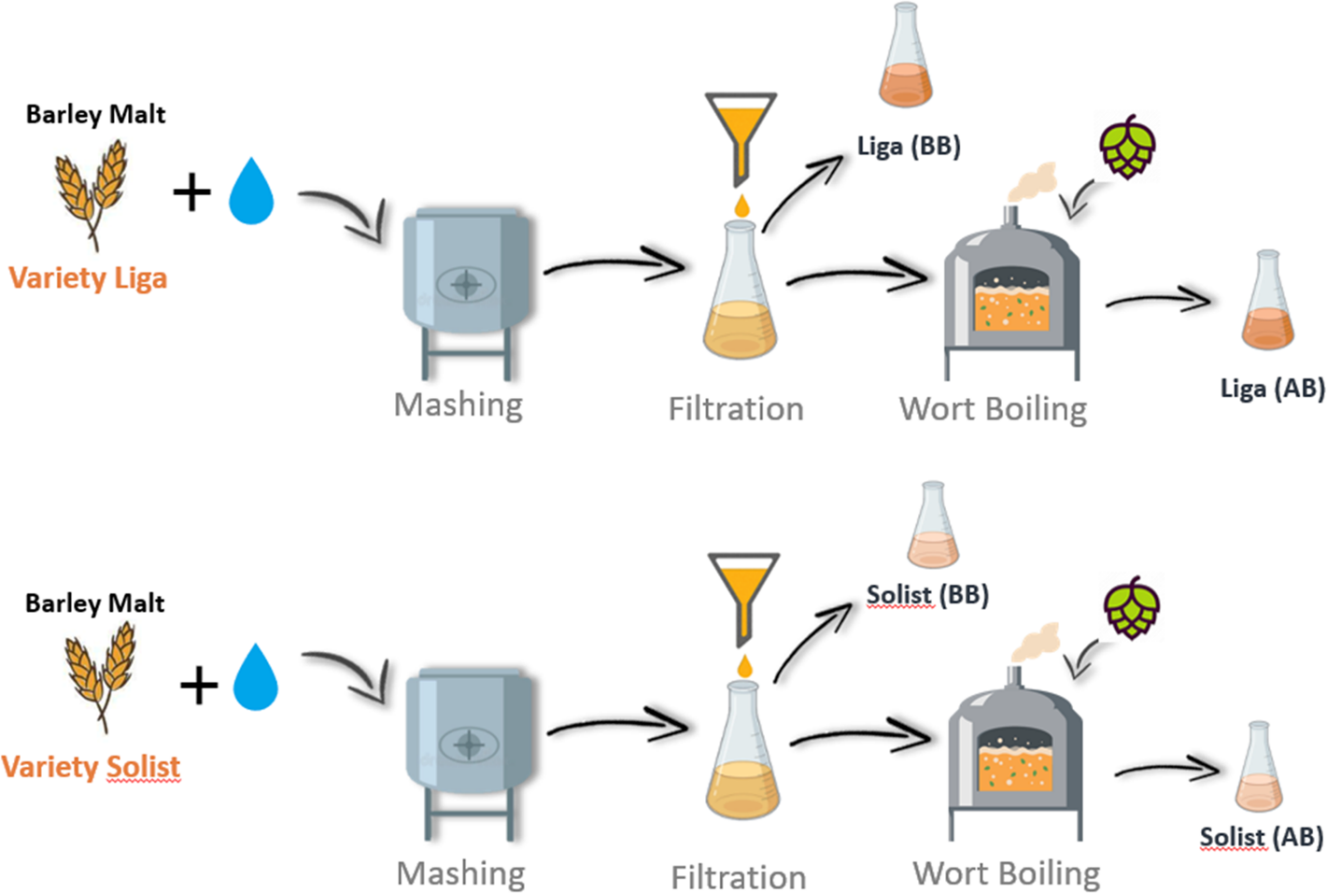
Design of experiment

For this study, experiments were designed (Figure 1) to explore the effect of commercial barley cultivars on hop bitter acid utilization. The wort was produced using 100% Pilsner barley malt from the Liga or Solist barley varieties (Ireks, Kulmbach, Germany) ground at 1.2 mm and mixed with distilled water (1:4 w/w). Those barley cultivars were chosen for this study based on their wider usage in the brewing industry and their proteome variation. This design of the experiment focused on comparing cultivars that were processed similarly into Pilsner malt. The same barley malt batch was used for all trials to avoid variability. The trials were conducted in triplicate, and the samples before boiling (Liga-BB/Solist-BB), Liga wort after boiling (Liga-AB), and Solist wort after boiling (Solist-AB) were collected for further analysis and characterization. The mashing was performed in a mash bath following the mashing scheme presented in Figure S1 (Supporting Information) which contains details of the time and temperature used. The sweet wort was separated from the brewer’s spent grain by filtration using a folded paper filter with a diameter of 320 mm (Whatman, Dassel, Germany). Hop CO_2_ extract obtained from Hopsteiner Company (Nürenberg, Germany) was added to achieve 90 mg/L of α-acids. Hop CO_2_ extract was used in this experiment to avoid the addition of hop polyphenols and, therefore, ensure a more controlled and consistent experimental setup. The boiling was done under reflux and lasted 60 min until the wort was cooled to 20 °C. Prior to the Liga-AB and Solist-AB sample collection, the wort was filtered to separate the trub formed during the boiling. The samples were then stored in a freezer at −18 °C until the analysis.

### Wort Characterization

Table 1 shows the analysis performed on the wort. The methodologies were according to the Mitteleuropäische Brautechnische Analysenkommission eV (MEBAK). The complete wort characterization data are present in Table S1 (Supporting Information).

**Table 1:** Wort Characterization Methodologies According to MEBAK

analysis	MEBAK method	MEBAK online ref
density^a^	B-590.08.904	(15)
real extract^a^	B-590.10.181	(16)
apparent extract^a^	B-590.09.900	(17)
pH^a^	B-590.00.040	(18)
original gravity^a^	B-590.10.181	(16)
calorie^a^	B-590.78.999	(19)
total nitrogen	B-400.07.003	(20)
free amino nitrogen (FAN)	B-400.11.111	(21)
total polyphenols	B-590.41.111	(22)
β-glucan	B-400.26.900	(23)
bitter units (BU)	B-400.17.110	(24)

a Analyses performed on the density meter DMA 4500 M equipment (Anton Paar GmbH, Graz, Austria).

### Bitter Acid Analysis by HPLC

Hop α- and i*so-α*-acid homologues were analyzed according to ASBC-Methods of Analysis Beer 23-C in a high-pressure liquid chromatography (HPLC) system Agilent 1100 series HPLC system (Agilent Technologies, Böblingen, Germany) at a flow rate of 1.0 mL/min with a 5 μL injection volume. Two mobile phases were used. Mobile phase B was 100% methanol, and mobile phase A was 59% methanol, 40% water, and 1% phosphoric acid (85%). The elution began with 100% of mobile phase A for the first 28 min and changed to 100% B over the next 12 min. The last 15 min of analysis corresponded to 100% of phase A. The ASBC method was adapted regarding the mobile phase gradient, flow rate, and run time to improve the analysis of the equipment used. A Purosphere Star LC-18 5 μm silica column (250 mm × 4.6 mm, Merck, Darmstadt, Germany) was used for separation. Absorbance was measured at 270 and 314 nm. The international calibration standards ICS-I4 and ICS-A1 obtained from Labor Veritas (Zürich, Switzlerand) were used as standards for all measurements.

### Hydropathy and Isoelectric Point Determination

The grand average of the protein classes’ hydropathic value (GRAVY) profile was calculated by the gravy calculator (https://www.bioinformatics.org/sms2/protein_gravy.html) using the protein sequence in FASTA Format obtained from the Uniprot database. The isoelectric point (p*I*) was calculated using the isoelectric point calculator (https://www.bioinformatics.org/sms2/protein_iep.html).^25^

### Protein Extraction and Digestion

Wort proteins were extracted by acetone precipitation due to high efficiency, removal of contaminants, simplicity, and speed. Then 200 μL of wort was transferred to an Eppendorf tube, and 800 μL of cold acetone (at −20 °C) was added. It was incubated for 90 min at −20 °C, followed by a 10 min centrifugation (Concentrator 5305 plus, Eppendorf, Germany) at 13 000*g*. The supernatant was removed, and the pellet was dried for 30 min at room temperature. The protein pellets were resuspended with SDS sample buffer and boiled for 5 min at 95 °C. The samples were centrifuged at 17 500*g* for 10 min. The proteins were separated by SDS-PAGE gel using a Tris-Glycine SDS running buffer and a molecular weight marker to verify the protein separation. The gels were stained with colloidal Coomassie Blue followed by overnight washing. To the gel pieces were added 250 μL of 50 mM ammonium bicarbonate (ABC) before shaking for 30 min at 37 °C. The gel washing was performed with the subsequent addition of acetonitrile (ACN) and 50 mM ABC solution. The protein reduction took place in a shaker at 37 °C for 30 min with 150 μL of reduction buffer (10 mM dithiothreitol, DTT, in 50 mM ABC solution). This was followed by the addition of 150 μL of alkylation buffer (55 mM iodoacetamide, IAA, in 55 mM ABC Puffer) and incubated in darkness at room temperature for 20 min. The alkylation buffer was removed, and 150 μL of ACN was added to shrink the gel pieces for 5 min. The digestion was performed with the addition of 150 μL of trypsin buffer (0.005 μg μL^–1^ trypsin in 50 mM ABC, 5% ACN (v/v)), and the digestion proceeded overnight at 37 °C. The digestion was terminated by acidification with 6 μL of 10% trifluoroacetic acid (TFA), shaking for 15 min.

As described by Rappsilber et al. (2003), peptides were eluted from the StageTips with 2 × 10 μL of 80% acetonitrile and 0.1% TFA. The samples were dried by vacuum centrifugation (Concentrator 5305 Plus, Eppendorf, Germany) to be dissolved in 10 μL of 1.6% acetonitrile and 0.1% formic acid (FA) subsequently.

### Protein Identification

Liquid chromatography coupled with mass spectrophotometer (LC-MS/MS) analyses were performed on a Thermo Scientific Q Exactive HF hybrid quadrupole–Orbitrap mass spectrometer coupled online to Ultimate 3000 RSLCnano Systems (Dionex, Thermo Fisher Scientific). The analytical column was a temperature-controlled EASY-Spray C18 LC column with a particle size of 2 μm and a length of 500 mm (Thermo Fisher Scientific, Germany) operated at 45 °C. Mobile phase A consisted of 0.1% FA in water; mobile phase B consisted of 80% acetonitrile and 0.1% FA.

Peptides were loaded onto the column and eluted at a flow rate of 0.3 μL/min. Peptides were separated by a series of linear gradients: 2% to 38% buffer B in 40 min, 38% to 52.5% in 4 min, and then 90% in 1 min. Eluted peptides were ionized by an EASY-Spray source (Thermo Scientific, Germany) and introduced directly into the mass spectrometer.

The MS data were acquired in data-dependent mode. MS1 spectra were recorded at 60 000 resolution (scan range 350–1400 *m*/*z*) to ensure less than 5 ppm error for precursor ions and a mass range of 700–8400 Da (approximately 7–84 amino acid residues). In each acquisition cycle, the 10 most intense peaks with a charge ≥2 were individually isolated with a 1.6 *m*/*z* window. The isolated ions were fragmented using higher-energy collisional dissociation (HCD) with stepped collision energy (27%, 29%, and 31%). The maximum injection time for MS1 scans was set to 50 ms and the automatic gain control (AGC) was set to 3.0 × 10^6^ ions. The MS2 spectra were recorded at 15 000 resolution to maintain less than 10 ppm error for fragment ions while minimizing scan time and maximizing the number of sampled precursors, maximum injection time of 80 ms, and AGC set to 1.0 × 10^5^ ions. Dynamic exclusion was enabled with a single repeat count and 60 s exclusion duration. Parameters related to hardware performance, such as injection times and automatic gain control (AGC) settings, were configured according to the optimal settings recommended by the manufacturer.

### Data Processing

The unsupervised analysis using principal component analysis (PCA) was performed in R (package: FactomineR and ggplot) to assess the groupings or trends in the data. The data was automatically standardized by default of the PCA function from the FactorMineR package. Each variable was centered and scaled to have a mean of zero and a standard deviation of one before PCA was conducted. The scores and loadings plots were used to determine the number of principal components (Supporting Information, Table S2). Protein identification was conducted using MaxQuant^26^ version 1.6.12.0. The mass spectrometry data was searched against reference proteome: UP000011116 (downloaded from Uniprot). The default parameters in MaxQuant were applied. Label-free quantification was performed with at least two peptides per protein. The final data set contained the proteins quantified in three replicates of each treatment. The data was pretreated by filtering the noise and log_2_ transformation in Perseus 1.6 software.^27^ Each protein was analyzed by a student’s *t* test using Perseus’s default parameters. The resultant matrix was used for data preprocessing in Microsoft Excel (Table S2 in Supporting Information Excel file). The missing values were imputed by the average and the LFQ intensity was adjusted based on the injection ratio. The enrichment analysis was conducted in Microsoft Excel with the protein classes matching the gene ontology (GO) terms extracted from the Uniprot database. The relative abundance of each GO term was transformed in percentage to highlight the most prominent GO terms.

## Results and Discussion

### Effect of Barley Cultivar on Wort Composition

**Figure 2:**
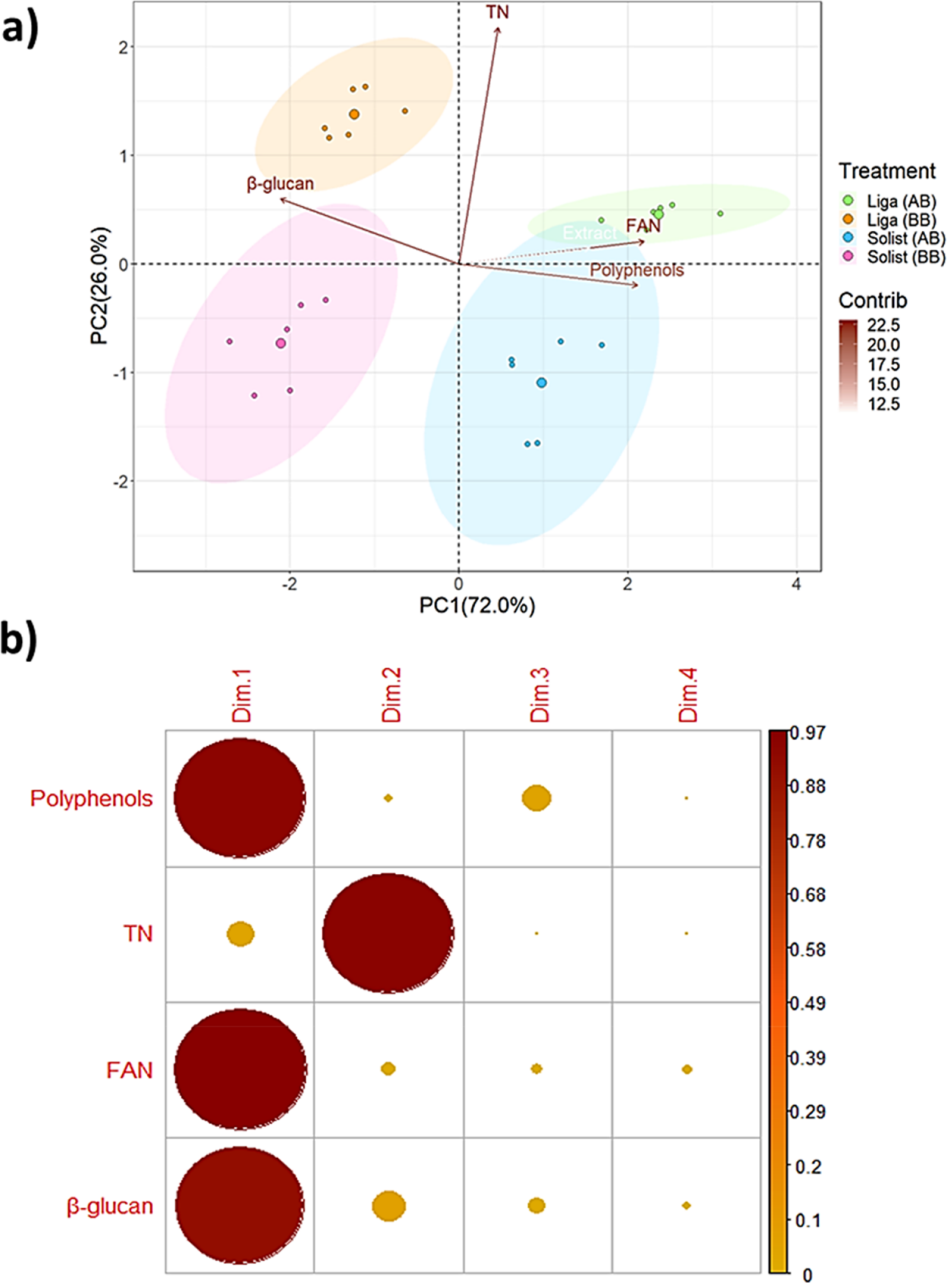
Wort composition showed in a principal component analysis: (a) PCA biplot of components 1 and 2 (the large sample dots represent the centroid of the ellipse, whereas the small dots represent each individual replicate. The “contrib” metric indicates how much each variable contributes to the formation of the principal components). (b) Correlation plot of variables in each dimension calculated by Cos^2^: Liga (BB), cultivar Liga before boiling; Liga (AB), cultivar Liga after boiling; Solist (BB), cultivar Solist before boiling; Liga (AB), cultivar Liga after boiling.

Barley malt, in addition to water, is the most prominent raw material in brewing by weight. It is essential in providing important macronutrients for fermentation performance and beer quality such as fermentable sugars, free amino acids, proteins, and polyphenols. The biplot graph presented in Figure 2a, resulting from the principal component analysis (PCA) offers a comprehensive visual representation of the relationships between variables and observations in the data set. The principal components (PC) were selected according to the eigenvalues for PC because the eigenvalues under 0.7 demonstrate lower significance.^28^ In this biplot, the principal components PC1 and PC2 collectively explain 97.94% of the total variance in the data set (Supporting Information, Table S2). The distribution of variables along PC1 indicates a strong positive correlation between free amino nitrogen (FAN) and polyphenols, while β-glucan appears to be negatively correlated with both FAN and polyphenols. Furthermore, total nitrogen (TN) is strongly positively correlated with PC2. Also, Figure 2b shows the correlation between variables and dimensions (PCs). This insight into variable relationships and their contributions to principal components facilitate a deeper understanding of the inherent structure within the data set and aid in identifying key variables driving the observed pattern.

The direction, magnitude, and color of the variable vectors in the biplot elucidate their contribution to the principal components. For instance, free amino nitrogen (FAN) appears to have the most significant influence on the direction of PC1, followed by polyphenol. Conversely, β-glucan has a minor impact on the orientation of PC1. Observations clustered toward the positive end of PC1 exhibit a higher correlation with FAN and polyphenols, represented by the post-boiling samples. FAN is normally formed during malting and mashing and its content in wort plays an important role in the fermentation performance of yeast by offering nutrition.^29^ Lower levels of FAN during fermentation can impact beer quality due to higher diacetyl production during fermentation.^30^ Surprisingly, the samples prepared with Solist cultivar were highly correlated with FAN. This cultivar protein profile might lead to an increase in FAN content during malting due to more available protein for proteolysis, although it contains lower soluble protein content. Furthermore, a statistically significant (*p* < 0.05) decrease in FAN content in the samples after boiling (AB) in comparison with before boiling (BB) was observed (Supporting Information, Table S3). This confirms the phenomenon observed in the previous study which demonstrated the reduction of FAN content in wort boiling due to Maillard reaction by melanoidin formation as well as precipitation to the trub.^12^

The Solist cultivar also showed a stronger correlation with the polyphenol content, as evidenced by the proximity of the clusters to this variable. Polyphenols typically originate from malt or hops. However, in this experiment, only hop extract, which contains no polyphenols, was used.^31^ Therefore, it can be inferred that the polyphenol content in these samples is directly related to barley malt polyphenols, which can significantly influence beer quality, particularly in terms of flavor and haze stability.^32^ Polyphenols contribute to trub formation by interacting with proteins from malt, forming colloidal particles that precipitate after boiling.^33^ The wort prepared with the Solist cultivar contained 20% more polyphenols than did the wort prepared with the Liga cultivar.

Additionally, the biplot reveals distinct clusters of observations based on their similarities and differences in the data set. As expected, the clusters of the Liga cultivar, located toward the positive end of PC2, comprise observations characterized by high values of TN. Because TN measurement demonstrates a great correlation with protein content, it can be seen that this barley cultivar contains greater protein content in comparison with the Solist cultivar even in the post-boil samples, demonstrating that the barley varieties differ greatly in protein content even during brewing.^34^ Furthermore, a clear reduction (*p* < 0.05) in TN of samples after boiling was observed. The precipitation of protein during boiling is paramount to improving beer quality regarding haze formation. Haze-active proteins interact with polyphenols during wort boiling, forming colloidal particles with a higher molecular weight, which, consequently, leads to precipitation to the trub.^35^ This haze-active protein removal avoids colloidal instability in beer during storage.^36^

These findings emphasize the necessity of controlling boiling conditions to optimize the utilization of hop bitter acids, thereby enhancing the final product’s quality. By elucidating the role of protein precipitation during boiling, this study provides a foundation for developing targeted strategies to increase hop bitter acid content, enhancing beer quality and bitterness. The significant reduction in total nitrogen (TN) post-boiling highlights the role of boiling in removing proteins that might interact with those compounds, consequently, carrying them to the trub. These findings shed light on brewing practices, allowing for the optimization of boiling conditions and the selection of barley cultivars that contribute to improved hop utilization in brewing.

### Barley Cultivar Protein Profile

**Figure 3:**
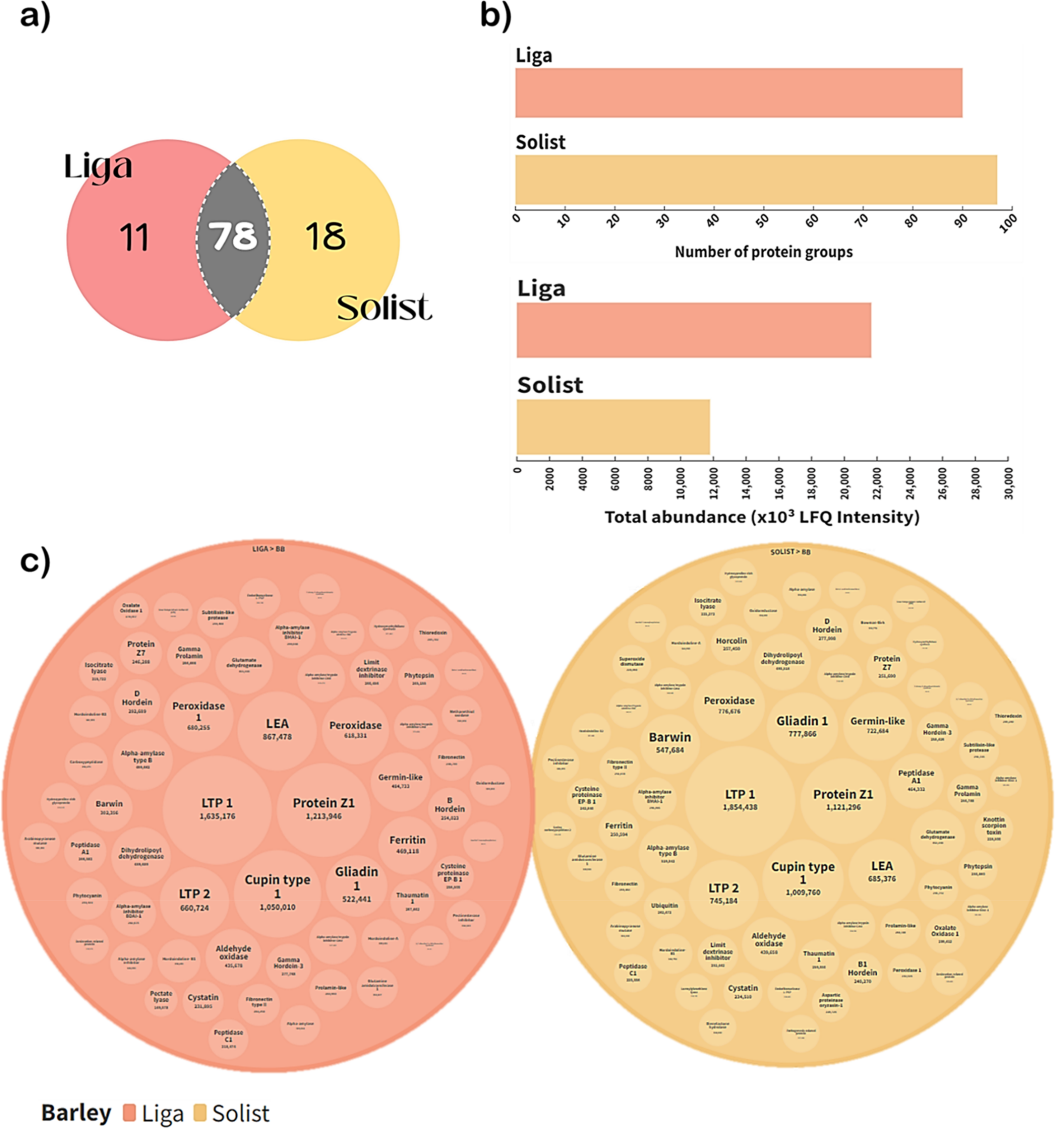
Barley cultivar protein profile: (a) Venn diagram representation of the number of proteins of each cultivar and in common among them. (b) Bar plot with the total number of proteins and total abundance in LFQ Intensity for each cultivar. (c) Tree map representation of protein families found in each cultivar with the respective LFQ Intensity.

Barley typically comprises 8–15% protein, with variability influenced by factors such as cultivar type and growing conditions. As mentioned, this research utilized malt from two distinct German barley cultivars, namely, Liga and Solist, to produce wort. The proteomic analysis of the wort aimed to characterize its overall protein composition. Initially, 118 protein groups were identified, but 11 were considered noise as they were detected exclusively in the post-boiling sample. Thus, the resulting data set comprised 107 protein groups (Table S4 in the Supporting Information Excel file). Among these, 78 protein groups were shared between both cultivars, as shown in the Venn diagram in Figure 3a. Additionally, 11 protein groups were unique to the Liga cultivar, while 18 were specific to the Solist cultivar. Interestingly, despite Liga having a total protein abundance in LFQ intensity twice that of Solist, it contained a lower number of identified proteins (Figure 3b).

These findings suggest that the genetic characteristics of each barley cultivar led to distinct protein expression profiles, which, in turn, impact the brewing process and final beer quality. For the Liga cultivar, proteins such as α-amylase inhibitor, B hordein, carboxypeptidase, ferritin, and peroxidase 1 are unique. These proteins play distinct roles in the brewing process. For instance, α-amylase inhibitors can affect the breakdown of starches, while ferritin and peroxidase 1 are involved in oxidation–reduction reactions which can influence beer stability and flavor.^37−39^ In contrast, the Solist cultivar has unique proteins, such as aspartic proteinase oryzalin-1, B1 hordein, barwin, bowman–birk, gliadin 1, and ubiquitin. Bowman–birk inhibitors are known to affect proteolytic activities, which can influence protein stability and clarity in beer. This highlights the role of cultivar genetics in protein variability, in this case, for water-soluble protein.

Illustrated in Figure 3c is a circle chart showing the distribution of the protein families. Notably, the predominant protein families identified are LTP and protein Z, recognized for their contribution to beer foam stability.^40^ These proteins constitute a significant portion of the water-soluble fraction in wort, as confirmed in a previous investigation.^12^ While LTP, protein Z, cupin type, and peroxidases emerge as the most abundant protein families in similar proportions for both cultivars, the specific isoforms contributing to these abundances differ substantially between them. Despite the shared presence of many protein isoforms across barley cultivars, their ranking varies, highlighting distinct water-soluble protein profiles (Table S4 in the Supporting Information Excel file).

Genetic variation among cultivars is a well-known biological phenomenon that plays a crucial role in the barley proteome. This variation is significant during grain development and at later stages during malting.^41^ Inside cells, genes encode proteins, directly influencing their amino acid sequence and primary structure.^42^ Each protein family contains multiple isoforms with differing primary structures, indicating that they were encoded by distinct genes. Therefore, the barley cultivar’s genotype directly impacts protein profiles, particularly regarding protein isoforms. The findings of this study validate this phenomenon by highlighting differences in the proteome of the examined barley varieties. Researchers have extensively explored this variation over the past decades, emphasizing its importance in barley breeding programs and the malting process.^43−47^ For example, Wang et al.^43^ conducted a proteomic analysis of two hulless barley cultivars, identifying 4603 proteins, with 326 showing differential expression between the cultivars.

Environmental factors, including growing conditions and agricultural practices, also play a significant role in shaping the protein profiles of barley cultivars. Aside from the genetic variation among barley cultivars, the proteomic differences observed in this study can be partly attributed to environmental factors. This was evidenced by Halstead et al.^48^ study, which conducted a comprehensive evaluation of five fall-planted malting barley lines across three distinct locations in the Pacific Northwest, each representing a unique growing environment. Notably, the nitrogen treatment (N_2_) that included an extra application at the heading led to an increase in the level of grain protein. Another study conducted by Luo et al.^49^ highlighted the role of nitrogen fertilizer and the environmental impact on the barley protein profile, although the study suggests that the genetic variation of the plant has a more significant impact on the protein content and proteome of malt.

**Figure 4:**
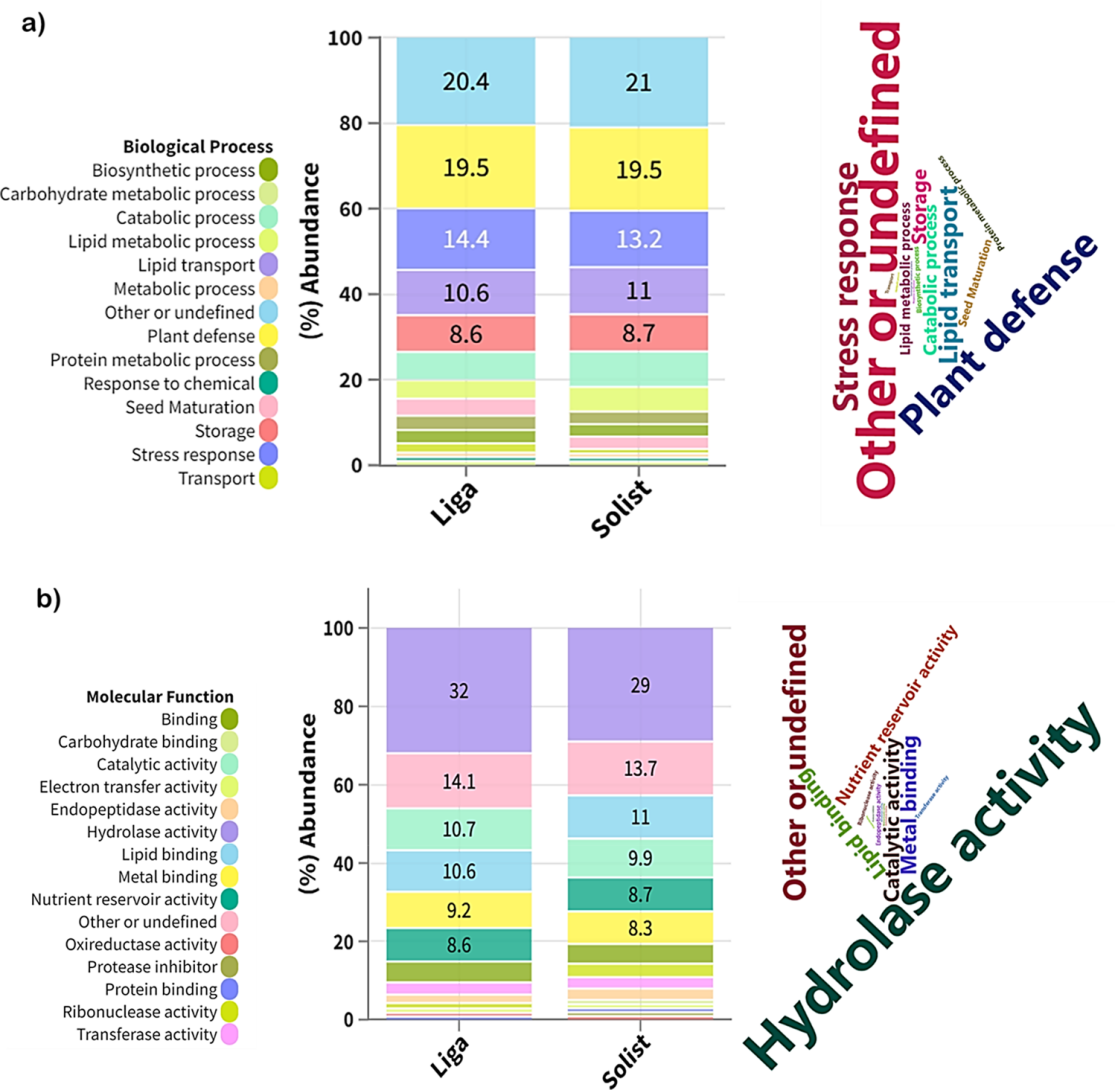
Barley cultivar biological protein profile: (a) biological process of proteins represented in a word cloud and a stacked bar plot in percentage. (b) Molecular function of proteins represented in a word cloud and a stacked bar plot in percentage.

Proteins are characterized as polypeptides fulfilling biological roles. In this investigation, they underwent annotation into gene ontology (GO) category enrichment analysis, aiding in the comprehension of proteome data, biological process (BP), and molecular function (MF) terms extracted from the UniProt database are used for annotating the proteins. The distribution of term frequencies among the 107 proteins in the samples is visualized in the word cloud presented in Figure 4. Nevertheless, due to incomplete entries in the UniProt database concerning barley proteins, a considerable portion of BP and MF is labeled as “other or unidentified”, constituting 21.5% and 15.0%, respectively, of the total proteins.

Notably, hydrolase activity emerges as the predominant molecular function, accounting for 30% of the frequency, indicating the prevalence of enzymes within the samples, representing nearly half (46.7%) of the proteins. This observation is expected due to enzymes’ higher solubility in aqueous media, as they require a certain level of water for their enzymatic activity and are likely to interact with surrounding water molecules.^50,51^ Furthermore, barley seed germination is induced during the malting process. In this step, enzymes are synthesized in the aleurone layer to degrade the nutrient reservoir and initiate the plant growth metabolism.^42^ For instance, in this study, a 6,7-dimethyl-8-ribityllumazine synthase enzyme was found. It is involved in the biosynthesis of riboflavin in plants and, consequently, essential for biosynthetic metabolism (Table S5 in the Supporting Information Excel file).^52^ In addition, metal binding was a significant molecular function, accounting for 11% of the frequency. These proteins may play an important role in metal chelation, potentially enhancing beer flavor stability by reducing the availability of metal ions in wort.^53^

Additionally, the word cloud in Figure 4a highlights that the most common BPs are plant defense and stress response, accounting for 30%. In a related study by Kerr et al.,^54^ the barley proteome was investigated under pathogen infection conditions, indicating modifications in the plant protein profile in response to fungal contamination. Remarkably, oxalate oxidase was found to be more abundant in infected seeds, and this protein was also present in both barley cultivars examined in this study. These findings further underline the environmental influence on the barley proteome, highlighting its crucial role in shaping brewing quality traits and the brewing process.

The column plot presented in Figure 4 provides a detailed breakdown of the percentage abundance (LFQ intensity) of BP and MF terms for each barley cultivar. Despite notable differences in protein profiles among these cultivars regarding their protein isoforms, there is a clear similarity observed in both the BP and MF categories. This similarity suggests that several proteins may acquire similar functions within the organism’s metabolism through distinct pathways that are intricately linked to their structural characteristics. In that sense, it is clear that the malting process plays a major role in determining the barley proteome, specifically due to the germination of barley seeds. As described in the study by Jin et al.,^55^ the proteome of green barley, i.e., barley seed before germination, varies noticeably, however, some differences disappeared after the malting process. Moreover, the main proteins involved in the variability across the cultivars are pathogen-related proteins and hydrolases, as shown in this study.

**Figure 5:**
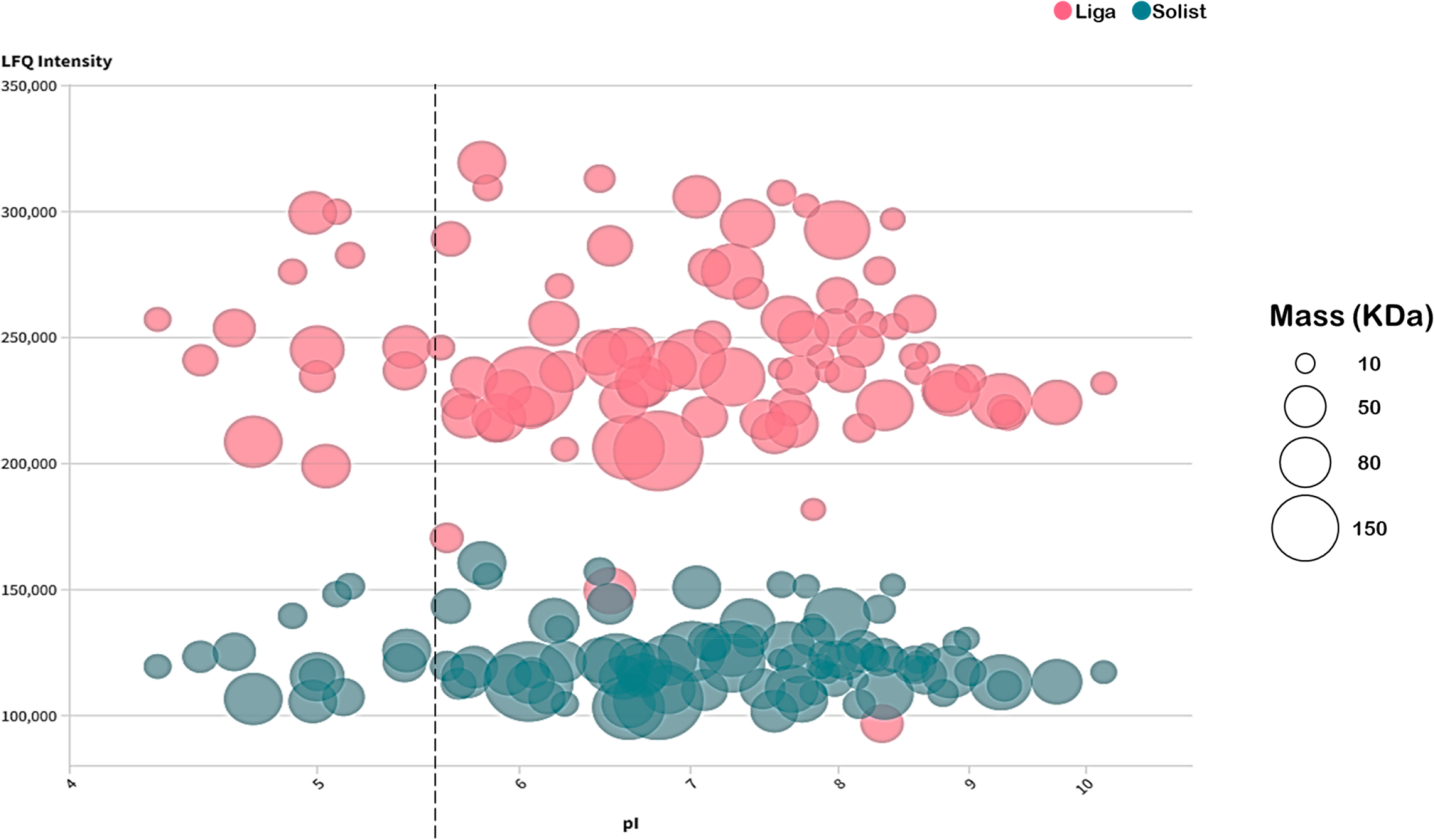
Barley cultivar protein chemical profile: bubble plot representation of the isoelectric point × LFQ intensity. The circle size represents the protein mass in kDa.

Each protein’s isoelectric point (p*I*) was determined using a bioinformatics tool that utilizes the FASTA index and protein sequence.^25^ This analysis aimed to explore the biophysical properties of water-soluble proteins sourced from different barley cultivars. The bubble chart in Figure 5 demonstrates the p*I* of proteins with the circle size indicating their molecular weight in kilodaltons (kDa). The distribution of proteins across the p*I* range reflects a typical pattern observed in biological systems, following a normal distribution with a peak in the neutral pI (Figure S5 in Supporting Information).^56^

The chart indicates a clustering of proteins with higher molecular weights around the neutral p*I*, suggesting that larger water-soluble barley proteins often possess a net-zero charge at neutral pH. Evidence supports the idea that larger proteins tend to exhibit a neutral or nearly neutral p*I*, whereas smaller proteins tend to show more extreme p*I* values.^57^ Despite the barley proteome reaching up to 9857470.16 kDa, the water-soluble proteins from the analyzed samples had <150 kDa mass due to mashing and filtration steps, separating the molecules with higher mass from the wort.^58^ For instance, both barley cultivars show two isoforms of aldehyde oxidase with the highest molecular weights, measuring 146.3 and 145.5 kDa, respectively. Conversely, the smallest proteins vary between cultivars, with 11.2 kDa (α-amylase/trypsin inhibitor CMc) for Liga and 8.8 kDa (Knottin scorpion toxin) for Solist, as detailed in Table S4 in the Supporting Information Excel file.

Interestingly, both cultivars share a similar p*I* range from 4.33 to 10.16, even with differences in the protein profile and protein distribution within this range. The Liga cultivar shows a more balanced distribution, with 52.8% of proteins falling in the alkaline range and 47.2% in the acidic range. In contrast, the Solist cultivar contains a higher proportion of alkaline proteins, with 56.2% in that range. Normally, the plant proteome comprises larger numbers of acidic p*I* proteins rather than basic p*I*. However, in this study, most of the proteins are found in the basic p*I* range due to the acidic pH of the wort. The protein’s solubility is usually lower at its p*I*, which explains the lack of acidic pI proteins in those samples.^59^

**Figure 6:**
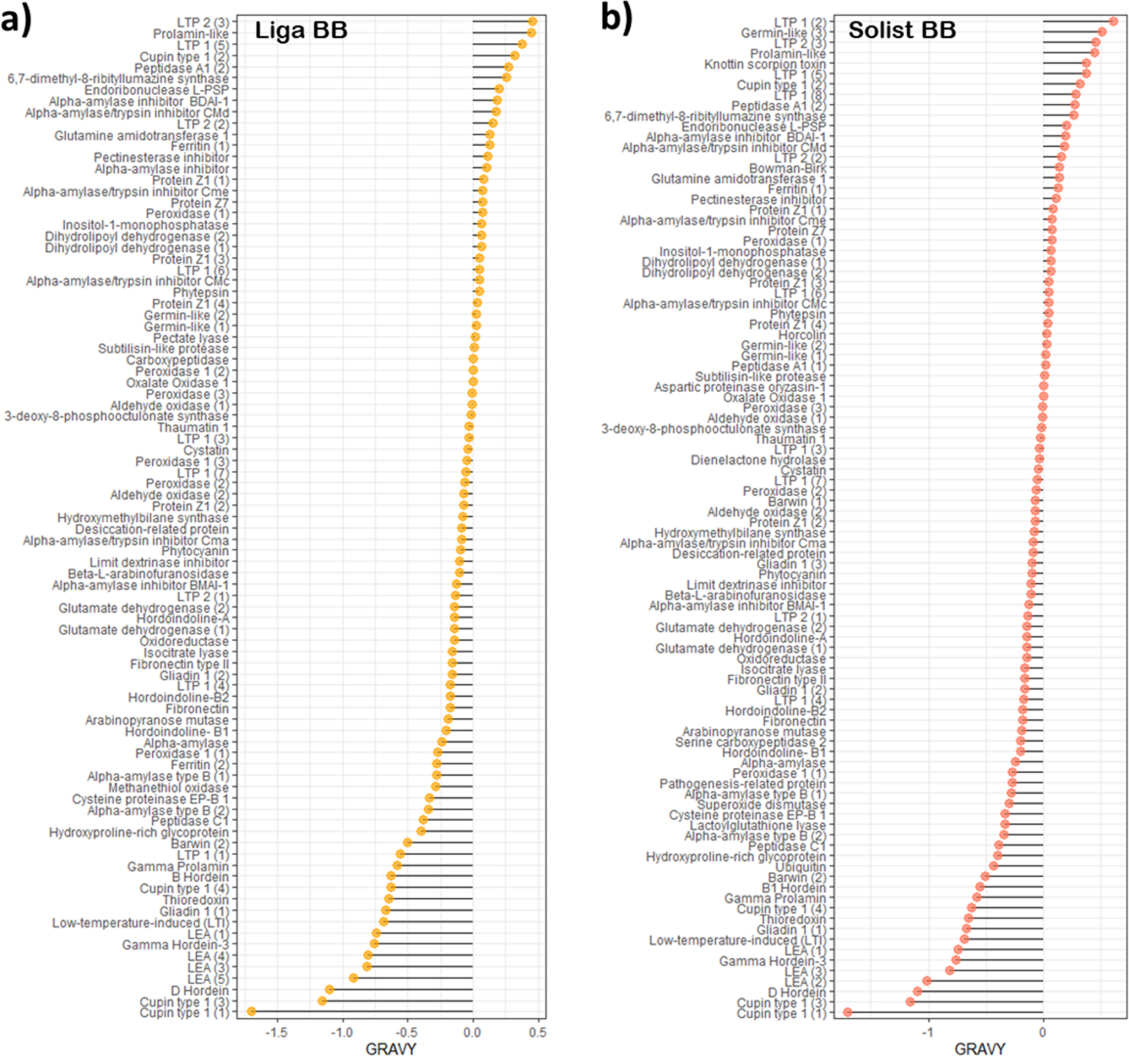
Barley cultivar chemical protein profile: lollipop chart of the grand average hydrophobicity (GRAVY) of (a) the cultivar Liga before boiling; (b) the cultivar Solist before boiling. The circle size represents the abundance in LFQ intensity of each protein.

Similarly to p*I*, the grand average hydrophobicity (GRAVY) index was computed using a bioinformatics tool that utilizes the FASTA index and protein sequence, revealing insights into the hydrophobic nature of the proteins (Figure 6).^25^ Positive GRAVY values indicate hydrophobic proteins, while negative values suggest hydrophilic proteins. Both barley cultivars predominantly exhibited proteins with negative GRAVY scores ranging from −1.7 to 0.0, consistent with previous findings from wort-boiled samples.^12^ A slight variance in hydrophobicity was observed between these cultivars, with Liga containing 65.56% hydrophilic proteins compared to 62.89% for Solist. This trend toward hydrophilicity within barley proteomics appears to be an inherent characteristic.^44^ In this study, this property is emphasized through the extraction of water-soluble proteins during the mashing step, facilitating the solubility of hydrophilic proteins.

### Effect of Barley Proteome on Hop Bitter Acid Utilization

**Figure 7:**
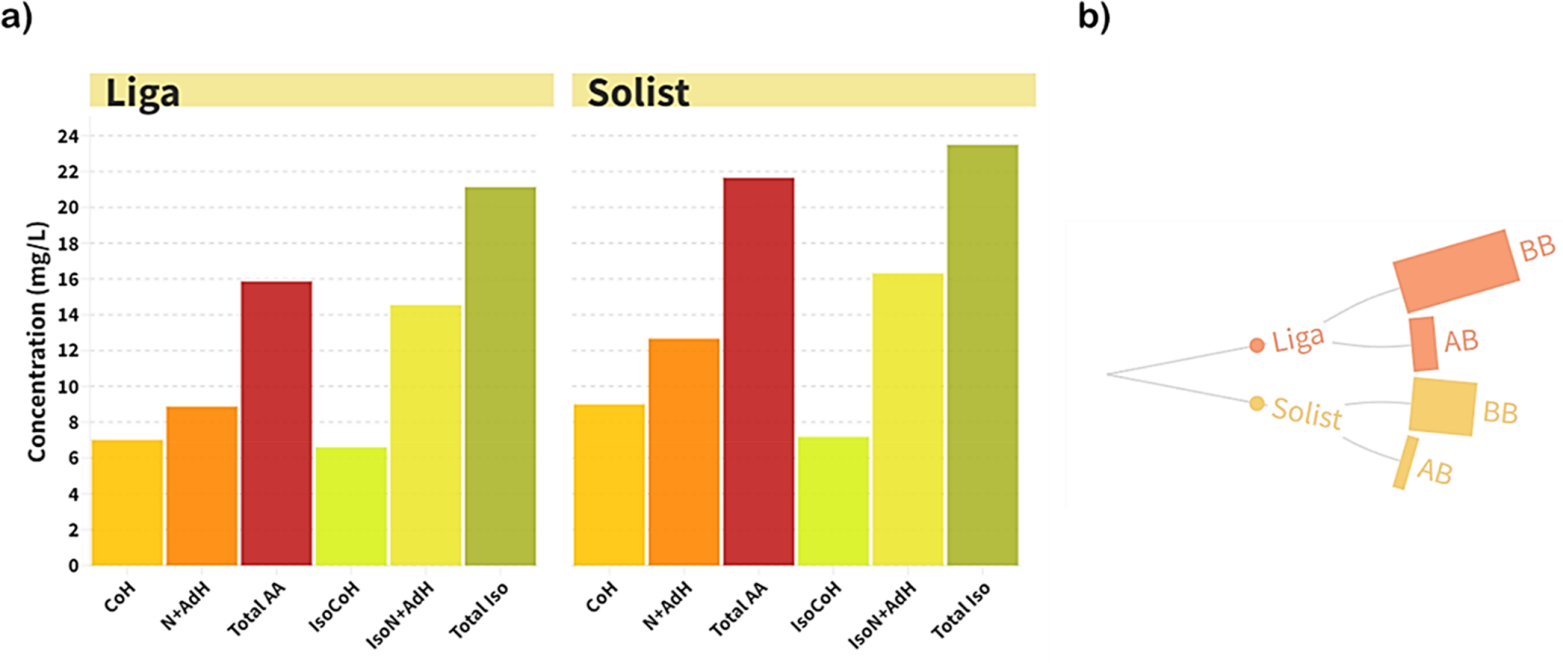
Protein precipitation and hop bitter acids: (a) hop α- and iso-α-acid homologue concentration in the hopped wort. (b) Bar plot representing the total abundance of proteins in LFQ intensity of each treatment; CoH, cohumulone; N + AdH, N– and adhumulone; total AA, total α-acids; iso CoH, isocohumulone; Iso N + AdH, *iso-*N– and *iso*-adhumulone; total iso, total *iso*-α-acids; BB, before boiling; AB, after boiling.

To investigate how different barley malt cultivars affect the utilization of hop bitter acids, two worts with malt from the different barley cultivars Liga and Solist were prepared, and hop CO_2_ extract containing 90 mg/L of α-acids in each one was added to reproduce typical brewing practices and industry standards to achieve a realistic concentration of *iso-*α-acids in the final beer. Further, hop bitter acid content in the samples post-boiling was analyzed by HPLC. The data are presented in the bar plot in Figure 7a. Clearly, the content of all homologues of α- and *iso*-α-acids, as well as the total content, was higher in the wort prepared with the Solist cultivar (*p* < 0.05). The wort prepared with Liga cultivar contained 15.86 mg/L of α-acids and 21.13 mg/L of *iso*-α-acids after boiling, whereas the wort produced from Solist cultivar had 21.65 and 23.49 mg/L, respectively.

To impart bitterness to beer, α-acids undergo a thermal isomerization reaction, converting into the more soluble *iso*-α-acid.^60^ This transformation is affected by various factors, particularly pH and original gravity.^61^ In this investigation, all worts were standardized to a pH of 5.6 and an original gravity of 16° Plato (Supporting Information, Table S1). Therefore, the only variable considered is the barley malt cultivar and, consequently, the barley proteome. The Liga cultivar exhibited a 45.51% higher total protein abundance (measured by LFQ intensity) compared with the Solist cultivar (Figure 7b). Additionally, wort derived from Liga showed a 10% reduction in *iso*-α-acid content post-boiling compared to that from Solist. This implies that the protein content and proteomic profile of the barley cultivar influence the precipitation of hop bitter compounds to the trub during wort boiling.

Protein aggregates primarily form during the heating-up phase and initial boiling, with a consistent particle size thereafter until boiling terminates.^62^ Intriguingly, this study observed a significantly higher difference in total α-acid content compared to *iso*-α-acids, showing a 26.75% higher in the wort produced from the Solist barley cultivar in comparison with the Liga cultivar (see Table S6 in the Supporting Information Excel file). This discrepancy suggests that either the protein profile or the protein content of barley malt directly influences the availability of α-acids for the isomerization reaction. It is well-known that the α-acids’ solubility is limited in aqueous solutions and acidic pH conditions, which can impact the rate of isomerization.^63^ To eliminate this factor, as stated before, the pH of all wort samples was consistently maintained across the trials. Consequently, the reduced α-acid content and the utilization of wort prepared with the Liga barley cultivar may result from the interaction with proteins, leading to precipitation.

The interaction between hop bitter acids and proteins is revealed in various beer properties, including foam stability and haze formation.^64^ During colloid formation at the beginning of the boiling step, α-acids interact with denatured proteins, possibly forming cross-links via hydrogen bonds, metal chelation, or hydrophobic interactions.^65^ A higher protein content, as observed in the Liga wort, could intensify interactions between specific proteins and α-acids by greater accessibility of soluble protein, resulting in the encapsulation of these compounds and, consequently, limiting their potential for isomerization into *iso*-α-acids. This phenomenon mirrors a mechanism detailed by Jongberg et al.^66^ for protein–polyphenol complexation, where polyphenols react with cysteine proteins’ thiol groups exposed during unfolding, forming covalent bonds. Furthermore, the unfolding of proteins, notably LTP1, during wort boiling may expose hydrophobic sites, promoting noncovalent hydrophobic interactions with polyphenols. This analogous mechanism could apply to hop bitter acids, especially α-acids, binding to proteins and protein complexes, as discussed in previous research.^12^ However, this hypothesis requires confirmation in further studies.

**Figure 8:**
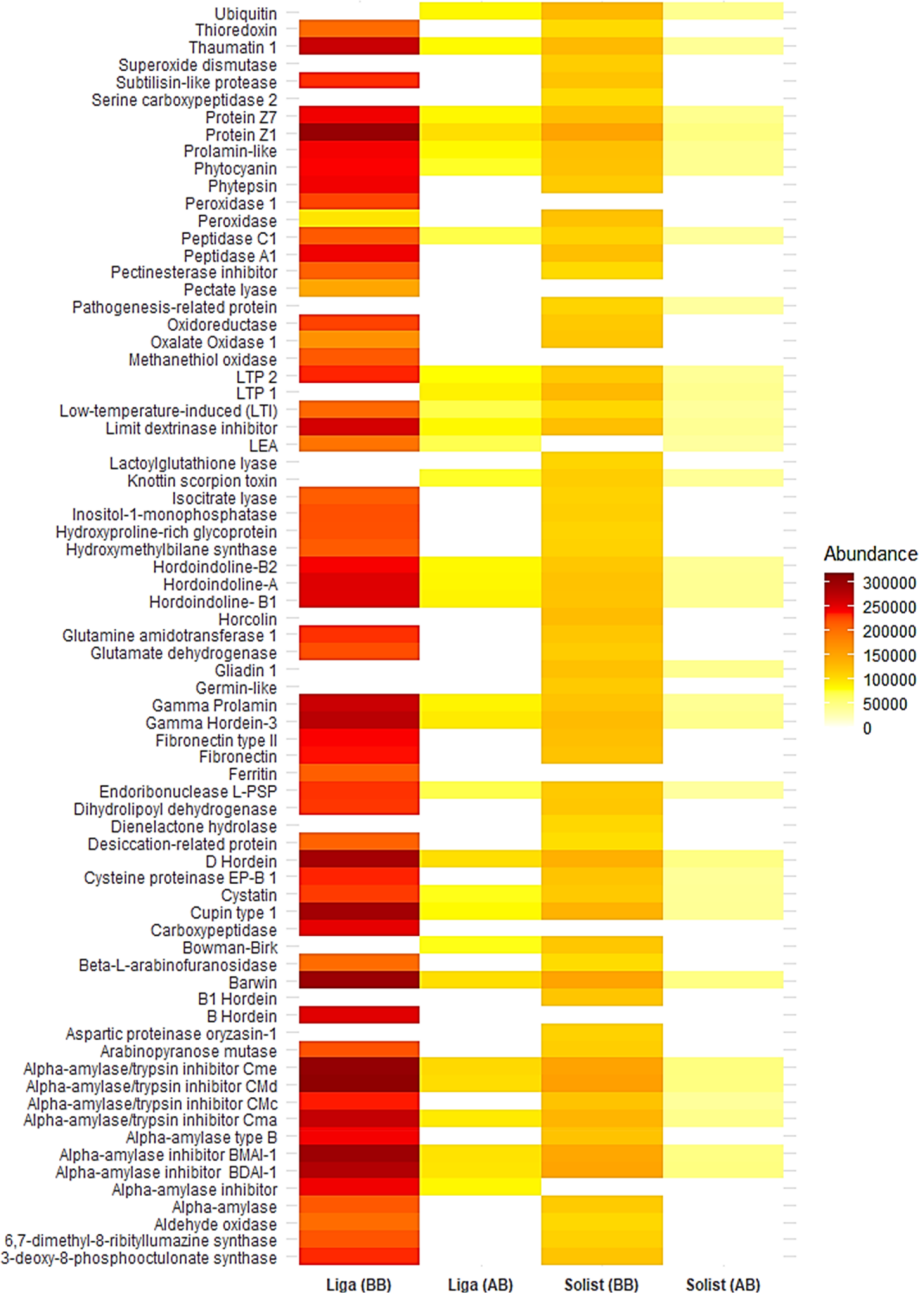
Heatmap plot representation of barley malt protein precipitation during the wort boiling step. For detailed information, see Supporting Information, Excel file.

Protein precipitation as trub is generally considered beneficial in brewing, enhancing beer quality by eliminating haze-active proteins.^67^ However, a significant amount of water-soluble protein ends up in this residue. Only one-fifth of the initial protein content was detected in the boiled wort prepared from the Liga or Solist cultivar, representing 21.47% and 19.16% of the total abundance (LFQ intensity), respectively. Figure 8 shows a heatmap presenting the individual protein abundances (LFQ intensity) for each sample. The 40 proteins in the wort from the Liga cultivar precipitated entirely due to boiling. From the Solist cultivar, 45 proteins precipitated after the same treatment. Of the precipitated proteins, both cultivars shared 32 proteins, primarily comprising enzymes like α-amylase, aldehyde oxidase, glutamate dehydrogenase, and thioredoxin. A notable observation was greater precipitation of higher molecular weight proteins (Supporting Information, Table S7). In the wort from the Solist cultivar, 51.11% of the total precipitated protein had a molecular weight greater than 40 kDa, compared to only 25.49% in the boiled wort. In contrast, the wort from the Liga cultivar exhibited an even higher proportion, with 65% of the total precipitated protein more than 40 kDa, in comparison to 24.49% of the soluble protein in the boiled wort. This trend was also observed in the study developed by Schultz et al.,^68^ which showed a considerable decrease in the abundance of high molecular weight proteins during wort boiling.

It is well-known that boiling wort induces protein unfolding via thermal denaturation, exposing inner hydrophobic sites that favor enthalpic interactions.^69^ Furthermore, evidence confirms that free cysteines are commonly surrounded by hydrophobic residues.^70^ Consequently, unfolded proteins interact via their hydrophobic sites and thiol groups, leading to the formation of protein–protein complexes. Notably, Jin et al.^71^ demonstrated the dependence of barley cultivars on the aggregation of unfolded proteins and chemical interactions, especially modification of sulfhydryl content. In this study, we observed a high degree of similarity in the protein profiles of precipitated proteins between cultivars, with the main discrepancies attributed to genotype-specific proteins. However, among the nongenotype-specific proteins, α-amylase/trypsin inhibitor CMc, cysteine proteinase EP-B1, and peroxidase 1 were exclusively precipitated in the boiled wort of the Liga cultivar. These proteins share a remarkable number of cysteine residues in their primary structure (Supporting Information, Figure S6), alongside hydrolase and binding activities. Additionally, Ilimure et al.^72^ suggested that α-amylase/trypsin inhibitor CMc is likely to precipitate with the trub, whereas in a previous study peroxidase emerged as an important protein likely to bind to hop bitter acids.^12^ Therefore, it is suggested that those proteins might play an important role in protein aggregation through the highly reactive thiol groups as well as the hydrophobic sites that might increase interactions among proteins and other compounds, such as polyphenols and hop bitter acids. While considerable attention has been paid to foam- and haze-active proteins in the brewing process, the current findings underscore the significance of minor proteins not directly linked to beer quality traits. Such proteins can indirectly influence process efficiency and flavor by forming interactions during boiling and further precipitation, thereby carrying important compounds, such as hop bitter acids. A previous study highlighted the specific proteins identified and their roles in the precipitation process and protein aggregate formation.^12^

**Figure 9:**
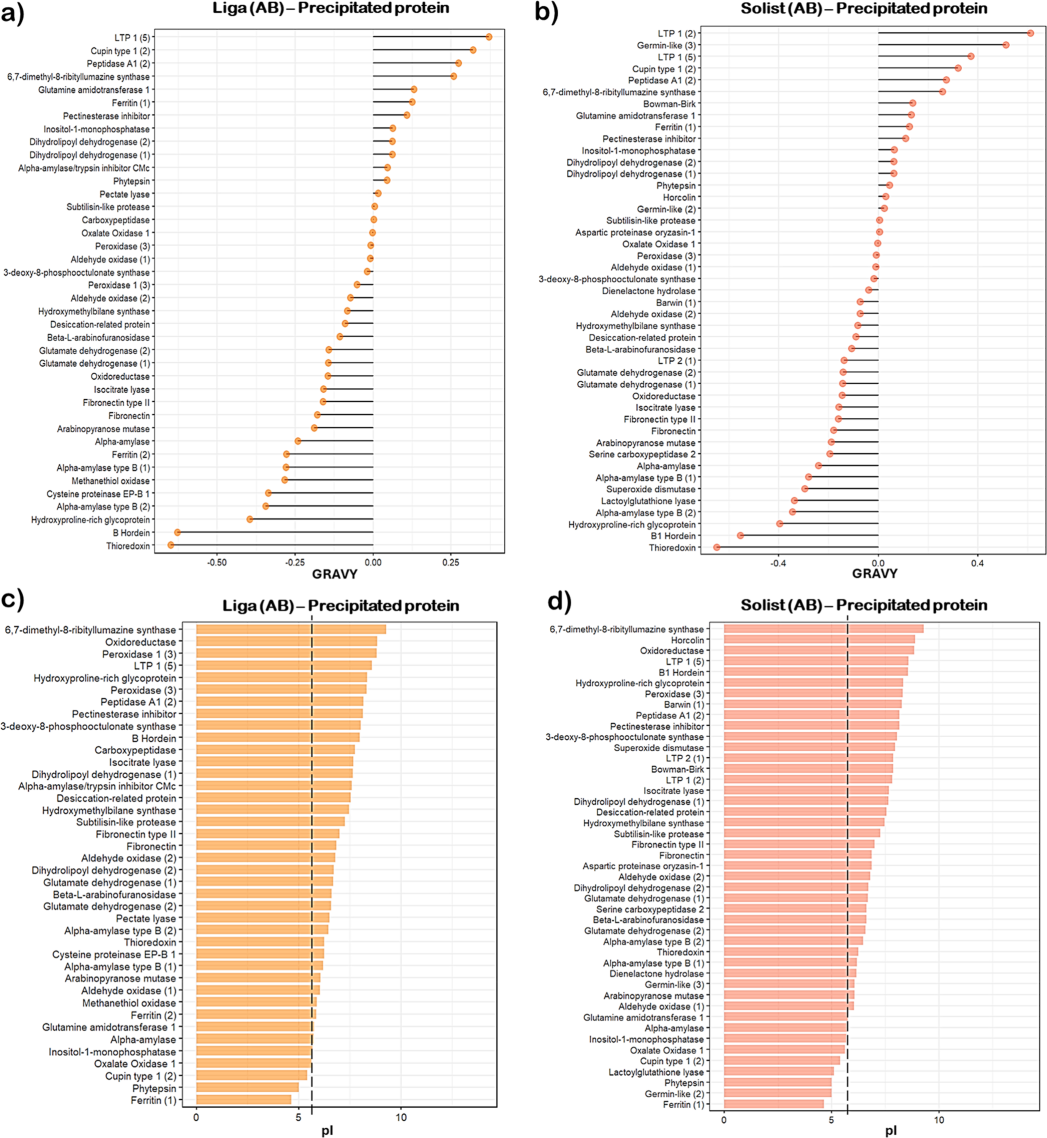
Physicochemical profile of protein completely precipitated during the boiling step: (a) Lollipop chart of the grand average hydrophobicity (GRAVY) of the precipitated protein of the cultivar Liga. (b) Lollipop chart of the grand average hydrophobicity (GRAVY) of the precipitated protein of the cultivar Solist. (c) Bar plot of the Isoelectric Point (p*I*) of the precipitated protein of the cultivar Liga. (d) Bar plot of the isoelectric point (p*I*) of the precipitated protein of the cultivar Solist.

To understand the physicochemical pattern of the entirely precipitated protein during the boiling step, GRAVY and p*I* values were determined as previously described. The results are presented in Figure 9. It is noteworthy that the observed trends in protein properties appear to be largely consistent across different barley varieties. The hydrophobicity (GRAVY) values of the precipitated proteins in the post-boiled wort show slight variations, ranging from −0.65 to 0.37 for the Liga cultivar and from −0.65 to 0.61 for the Solist cultivar. Notably, after boiling, the precipitated proteins tend to shift toward a higher hydrophobic range compared to their preboiling values (Figure 9a,b). The lowest value preboiling was −1.704 while after boiling stands at −0.65. This observation supports the idea that a protein’s primary structure, along with its physicochemical properties, can significantly influence its tendency to precipitate. Böhm et al.^73^ also found that hot trub contains elevated levels of hydrophobic amino acids. The author suggests that these higher levels of hydrophobic amino acids reduce hydration, affecting solubility and subsequently leading to aggregation through hydrophobic interactions postdenaturation. This phenomenon is evident in the findings of this study, which demonstrate a higher number of hydrophobic proteins that have been completely precipitated.

Protein p*I* is intrinsically correlated with protein solubility according to pH due to a neutral net charge of protein at pI. Once reaching this point, the protein experiences reduced hydration and electrostatic repulsion, leading to aggregation and subsequent precipitation via hydrophobic interactions.^59^Figure 9c,d illustrate the p*I* values of completely precipitated proteins for each barley cultivar. These p*I* values align closely with the wort pH of 5.6 (indicated by the dashed line). Surprisingly, the p*I* range (4.64–9.29) remains consistent across both barley varieties, suggesting that wort pH has a more significant impact on protein precipitation than the specific barley malt used. Protein precipitation by pH is widely used in the food industry and is also applicable in the brewing process, contributing to enhanced beer quality.^36^ In a study by Ilimure et al.,^72^ analysis of sweet wort, boiled wort, and trub revealed that proteins in the trub primarily fall within the p*I* range of 5.0–8.0. This study highlights the influence of barley genotype on the utilization of hop bitter acids, although the impact on protein precipitation is comparatively minor concerning physicochemical properties.

In summary, this study presented for the first time the influence of barley genotype on interactions between proteins during boiling and its subsequent effect on the hop bitter acid yield. By examining barley malt’s protein composition, genetic variation, and physicochemical properties, this research provides insights into how different barley cultivars affect the utilization of hop bitter compounds and protein precipitation during wort boiling. The findings suggest that barley cultivars influence the availability of hop bitter acids for isomerization, with implications for beer bitterness and quality. Additionally, the study highlights the role of interactions between proteins and physicochemical properties in protein precipitation, contributing to a deeper understanding of the brewing process and its environmental impact.

Based on the desired beer characteristics, brewers can select barley cultivars that align with their quality goals. For clear, stable beer, choosing a low-protein cultivar, such as Solist, might be advantageous. Conversely, if the goal is to enhance yeast fermentation performance, then a cultivar with higher FAN content should be considered. Brewers can optimize the boiling process to enhance protein precipitation. By closely monitoring and adjusting the boiling parameters to target protein precipitation, brewers can achieve better clarity and stability in their beer. Furthermore, the hop bitter acid utilization in brewing could be improved by the investigation of target proteins interaction and the chemical mechanism behind it.

Furthermore, additional studies are recommended to investigate the role of specific amino acids and protein domains in interactions with hop bitter acids. Future research could also be performed with additional commercial barley cultivars processed in different malt products to screen the role of barley genetic variation, malting process, and environmental conditions on hop bitter acids utilization.

## Supporting Information Available

Additional details of the methodology and data analysis (PDF)
Additional details of the proteomics data set (XLSX)

## Abbreviations

MEBAK: Mitteleuropäische Brautechnische Analysenkommission
HPLC: high-pressure liquid chromatography
GRAVY: grand average hydropathic value
p*I*: isoelectric point
SDS: sodium dodecyl sulfate
SDS-PAGE: sodium dodecyl sulfate–polyacrylamide gel electrophoresis
ABC: ammonium bicarbonate
ACN: acetonitrile
DTT: dithiothreitol
IAA: iodoacetamide
TFA: tridluoroacetic acid
FA: formic acid
LC-MS/MS: liquid chromatography coupled with mass spectrophotometry
MD: mass spectrometry
HCD: higher-energy collisional dissociation
AGC: automatic gain control
GO: gene ontology
PCA: principal component analysis
PC: principal component
TN: total nitrogen
FAN: free amino nitrogen
LFQ: label-free quantitation
LTP: lipid transfer proteins
MF: molecular function
BP: biological process
LEA: late embryogenesis abundant protein
