# Supplementary material for: The Influence of Barley Proteome on Hop Bitter Acid Yield during Brewing
Source: J Agric Food Chem. 2024 Sep 16;72(38):21166–80. doi: 10.1021/acs.jafc.4c04396 (PMC11440504; doi:10.1021/acs.jafc.4c04396)
Supplement: Supplementary file 1 — jf4c04396_si_001.pdf [file jf4c04396_si_001.pdf]

## Supporting Information for

# The influence of barley proteome on hop bitter acid yield during brewing

Mariana B. C. Pinto[ab], Flavio L. Schmidt[a], Zhuo Chen[c], Juri Rappsilber[c], Brian Gibson[b], Philip C. Wietstock[b]

[a] *Fruit and Vegetables Laboratory - Department of Food Technology, School of Food Engineering, University of Campinas (UNICAMP), R. Monteiro Lobato 80, 13083-862 Campinas, SP, Brazil*

[b] *Department of Food Technology and Food Chemistry, Chair of Brewing and Beverage Technology, Technische Universität Berlin, Berlin, Germany*

[c] *Bioanalytics, Institute of Biotechnology, Technische Universität Berlin, Berlin, Germany.*

*Correspondent author: philip.wietstock@tu-berlin.de

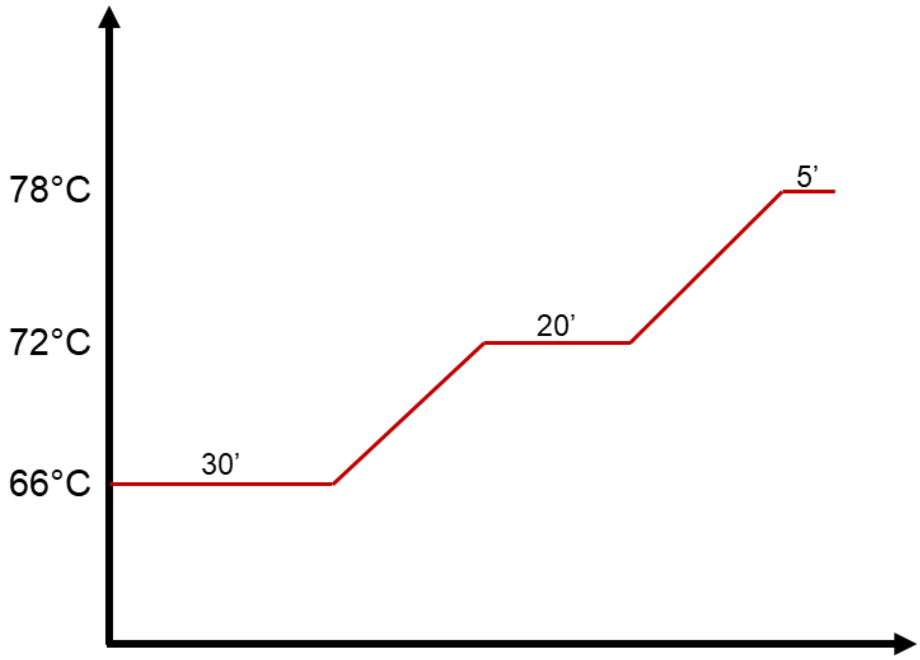


**Figure S1.** Mashing scheme parameters of time and temperature

**Table S1.** Wort characterization average values and standard deviation

| Sample | Liga (BB) | Liga (AB) | Solist (BB) | Solist (AB) |
|---|---|---|---|---|
| **Density (g/cm³)** | 1.066 | 1.066 | 1.066 | 1.066 |
| ***SD*** | *0.001* | *0.001* | *0.001* | *0.001* |
| **Er (Real extract) (% w/w)** | 16.66 | 16.67 | 16.39 | 16.52 |
| ***SD*** | *0.22* | *0.22* | *0.40* | *0.12* |
| **Ea (Apparent extract) (% w/w)** | 16.68 | 16.70 | 16.42 | 16.54 |
| ***SD*** | *0.21* | *0.22* | *0.41* | *0.12* |
| **p (Original gravity) (% w/w)** | 16.57 | 16.57 | 16.45 | 16.26 |
| ***SD*** | *0.23* | *0.25* | *0.39* | *0.11* |
| **pH** | 5.66 | 5.58 | 5.58 | 5.52 |
| ***SD*** | *0.01* | *0.02* | *0.00* | *0.01* |

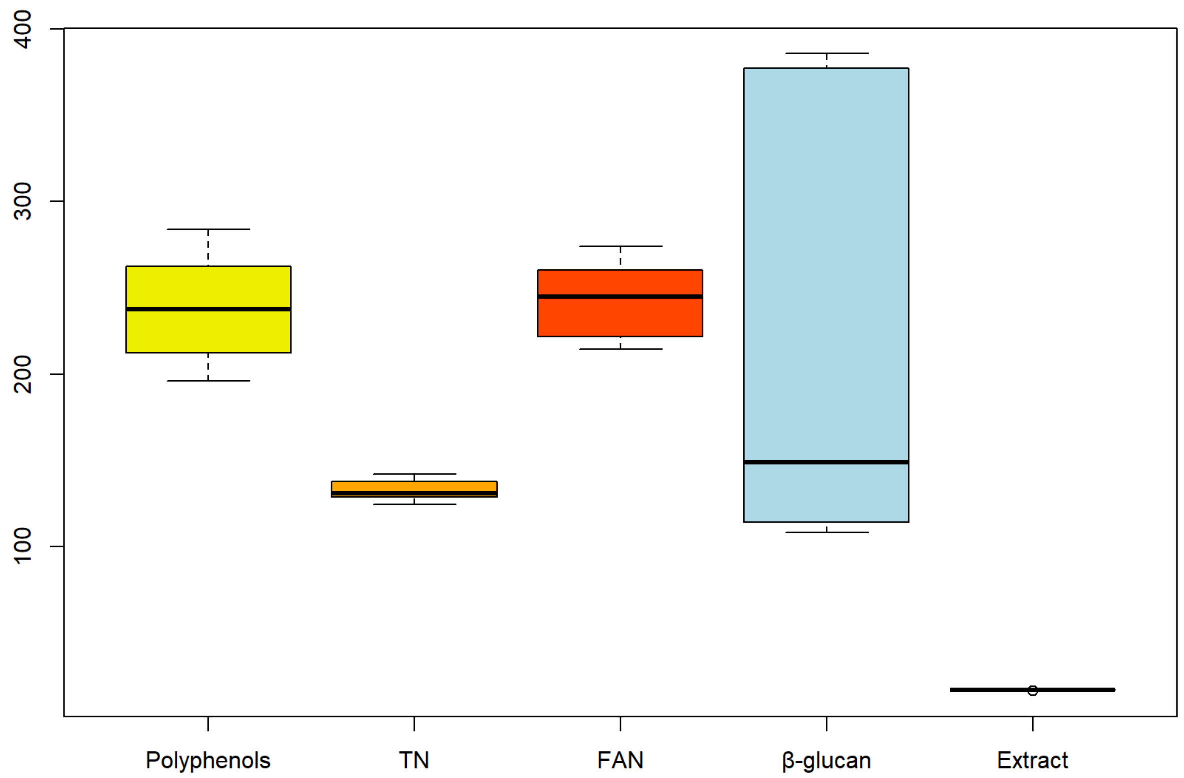


**Figure S2.** Boxplot analysis of wort composition results

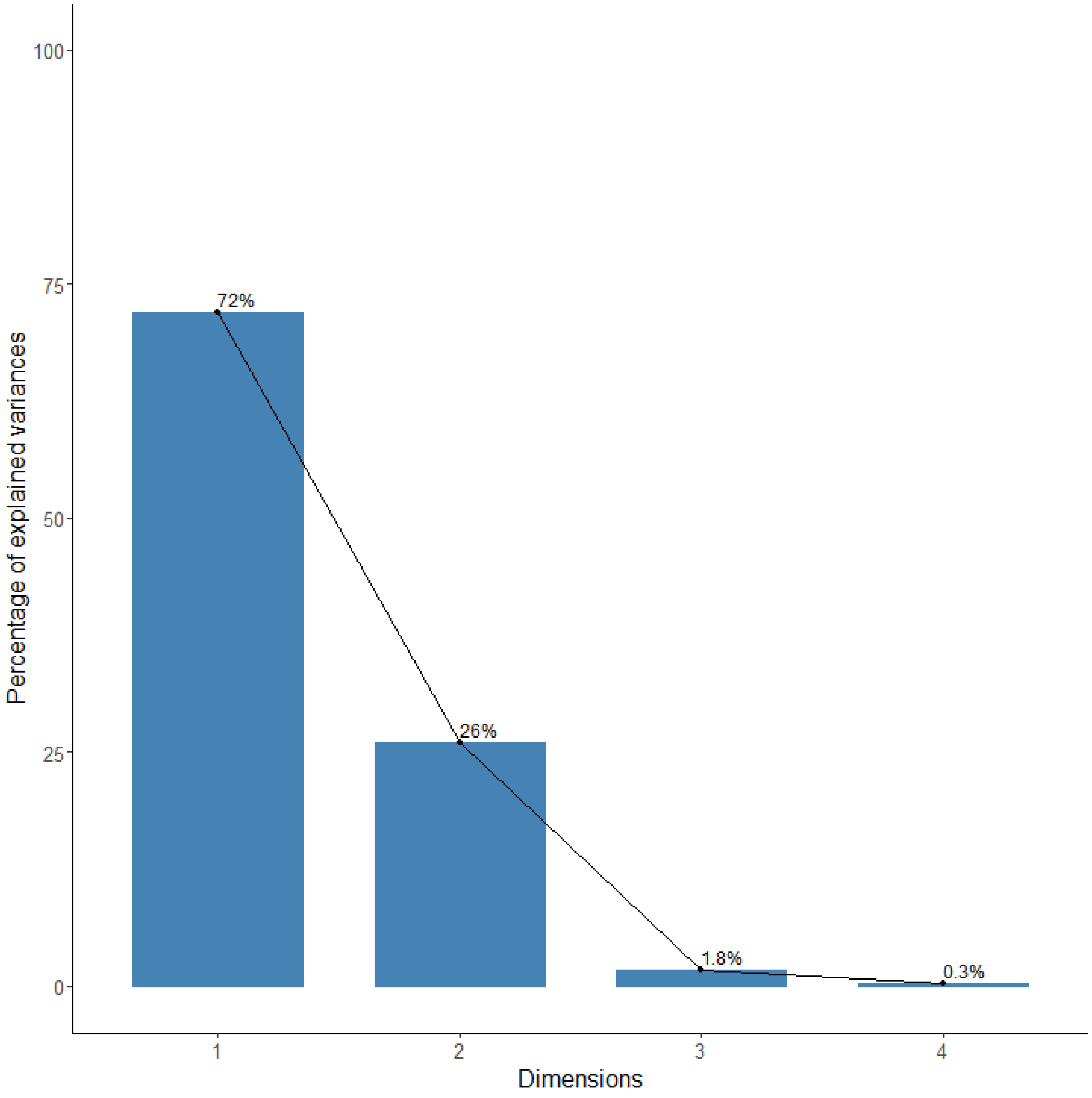


**Figure S3.** The proportion of variation of each dimension in percentage

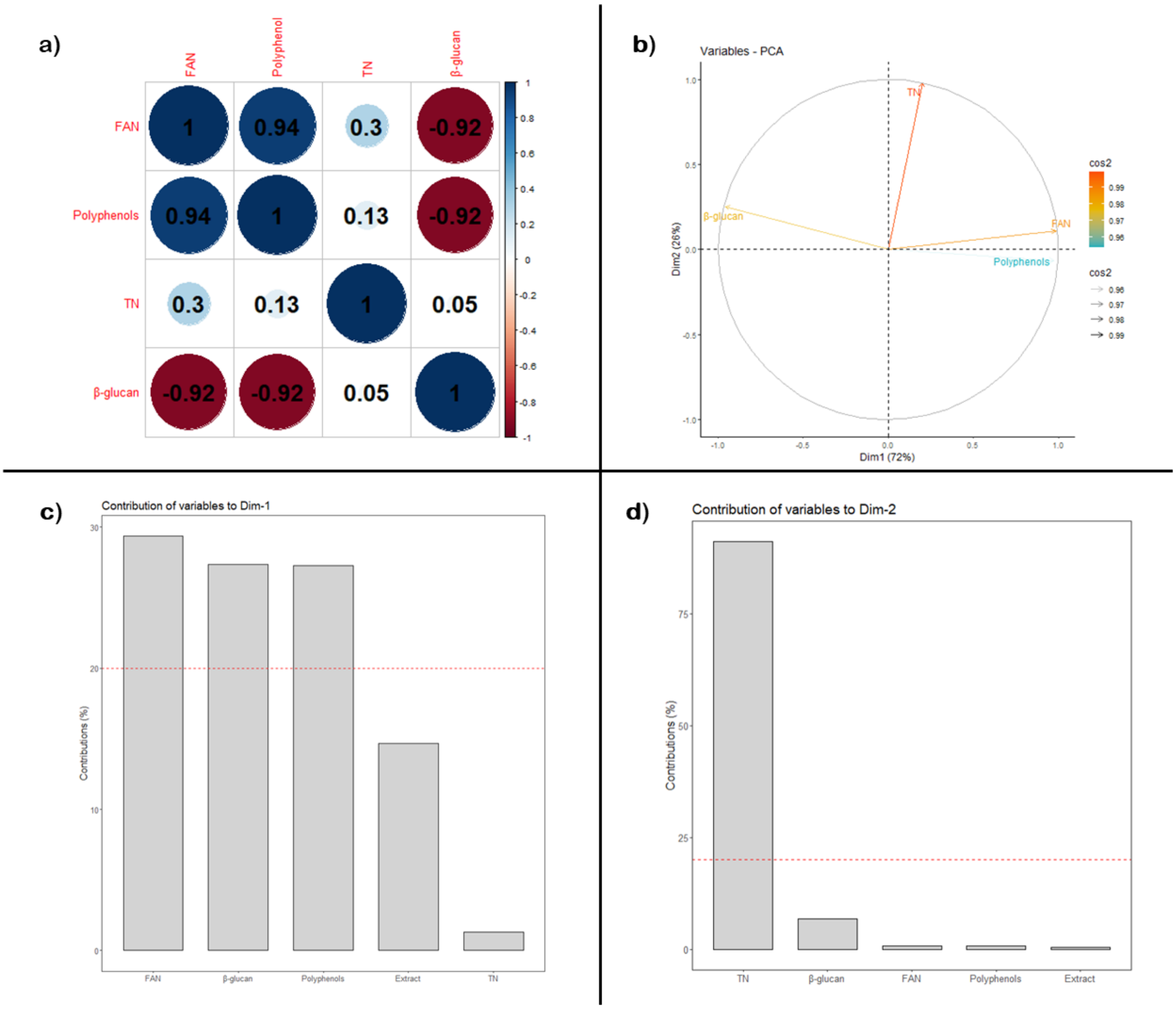


**Figure S4**. **(a)** Correlation Matrix of the wort composition parameters calculated by Peason's correlation; **(b)** Contribution of variables of principal component calculated by cos²; **(c)** Contribution of variables of principal component 1; **(d)** Contribution of variables of principal component 2

**The red dashed line on the graph indicates the expected average contribution*

**Table S2**. PCA discriminative values

| Component | Eigenvalue | Percentage of variance | Cumulative percentage of variance |
|---|---|---|---|
| **1** | **2.879** | **71.973** | **71.973** |
| **2** | **1.038** | **25.964** | **97.938** |
| *3* | *0.070* | *1.769* | *99.907* |
| *4* | *0.011* | *0.293* | *100.000* |

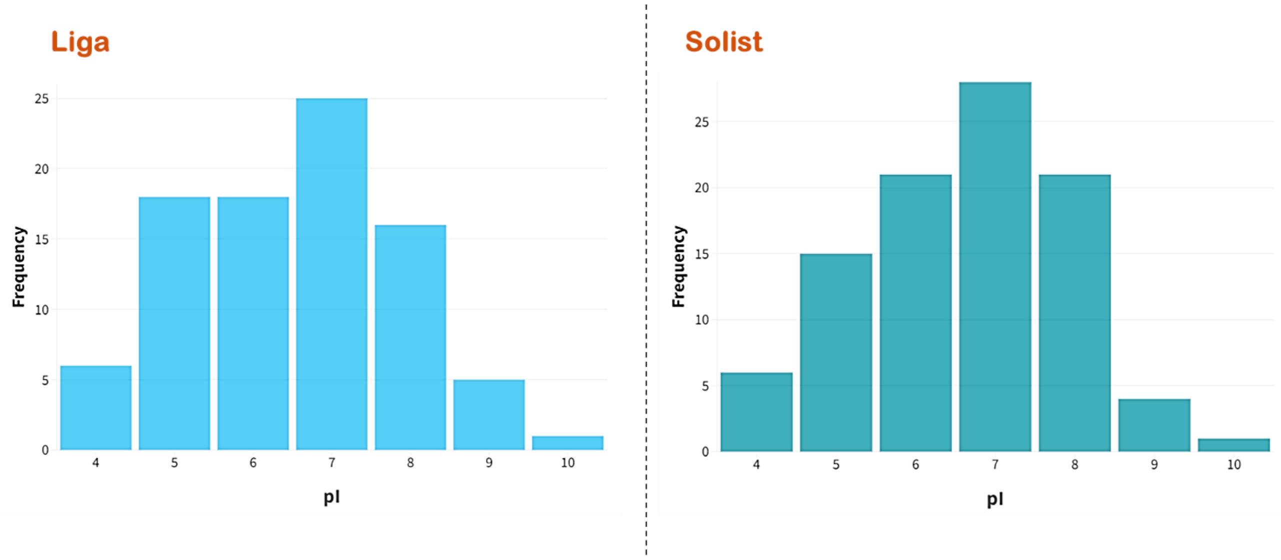


**Figure S5.** Protein pI histogram: Distribution of protein isoelectric point across the barley cultivars

**Table S3.** Dataset of wort composition

| Sample | Polyphenols (mg/L) | Average | SD | BU | Average | SD | Total Nitrogen (mg/100ml) | Average | SD | FAN (ppm) | Average | SD | β-glucan (ppm) | Average | SD |
|---|---|---|---|---|---|---|---|---|---|---|---|---|---|---|---|
| Liga (BB)<br>Liga (BB) | 223.7<br>224.9 | 224.3 | 0.9 | </= 0,1<br></= 0,1 | | | 140.0 | 140.3 | 1.2 | 227.4<br>227.0 | 227.2 | 0.3 | 385.1<br>385.6 | 385.4 | 0.3 |
| Liga (BB)<br>Liga (BB) | 212.0<br>213.7 | 212.9 | 1.2 | | | | 139.0 | | | 228.6<br>225.4 | 227.0 | 2.3 | 378.3<br>379.4 | 378.8 | 0.8 |
| Liga (BB)<br>Liga (BB) | 211.9<br>212.3 | 212.1 | 0.3 | | | | 141.8 | | | 233.1<br>232.6 | 232.8 | 0.4 | 363.0<br>363.8 | 363.4 | 0.5 |
| Liga (AB)<br>Liga (AB) | 217.6<br>217.6 | 217.6 | 0.0 | 30.2<br>30.4 | 30.3 | 0.1 | 128.5 | 127.4 | 2.3 | 215.6<br>214.2 | 214.9 | 1.0 | 384.5<br>383.4 | 384.0 | 0.8 |
| Liga (AB)<br>Liga (AB) | 195.7<br>199.1 | 197.4 | 2.4 | 33.2<br>32.2 | 32.7 | 0.7 | 124.3 | | | 214.8<br>214.8 | 214.8 | 0.0 | 377.1<br>377.2 | 377.1 | 0.1 |
| Liga (AB)<br>Liga (AB) | 208.7<br>208.3 | 208.5 | 0.3 | 27.8<br>27.9 | 27.9 | 0.1 | 129.5 | | | 218.2<br>217.4 | 217.8 | 0.6 | 376.6<br>376.4 | 376.5 | 0.2 |
| Solist (BB)<br>Solist (BB) | 283.6<br>282.0 | 282.8 | 1.2 | </= 0,1<br></= 0,1 | | | 137.2 | 137.3 | 0.2 | 273.9<br>273.5 | 273.7 | 0.3 | 109.8<br>108.0 | 108.9 | 1.2 |
| Solist (BB)<br>Solist (BB) | 257.6<br>257.2 | 257.4 | 0.3 | | | | 137.2 | | | 266.0<br>266.2 | 266.1 | 0.1 | 114.5<br>113.6 | 114.1 | 0.7 |
| Solist (BB)<br>Solist (BB) | 267.0<br>268.9 | 267.9 | 1.3 | | | | 137.6 | | | 266.2<br>266.4 | 266.3 | 0.1 | 114.0<br>108.6 | 111.3 | 3.8 |
| Solist (AB)<br>Solist (AB) | 271.5<br>270.2 | 270.9 | 0.9 | 35.0<br>35.3 | 35.2 | 0.2 | 130.6 | 128.0 | 2.5 | 253.9<br>254.1 | 254.0 | 0.1 | 108.6<br>146.1 | 127.4 | 26.5 |
| Solist (AB)<br>Solist (AB) | 237.6<br>238.7 | 238.1 | 0.8 | 35.4<br>35.2 | 35.3 | 0.1 | 128.8 | | | 244.6<br>246.4 | 245.5 | 1.3 | 148.8<br>134.2 | 141.5 | 10.3 |
| Solist (AB)<br>Solist (AB) | 254.5<br>257.2 | 255.9 | 1.9 | 34.3<br>34.6 | 34.4 | 0.2 | 124.6 | | | 246.4<br>246.9 | 246.6 | 0.4 | 135.3<br>137.7 | 136.5 | 1.7 |

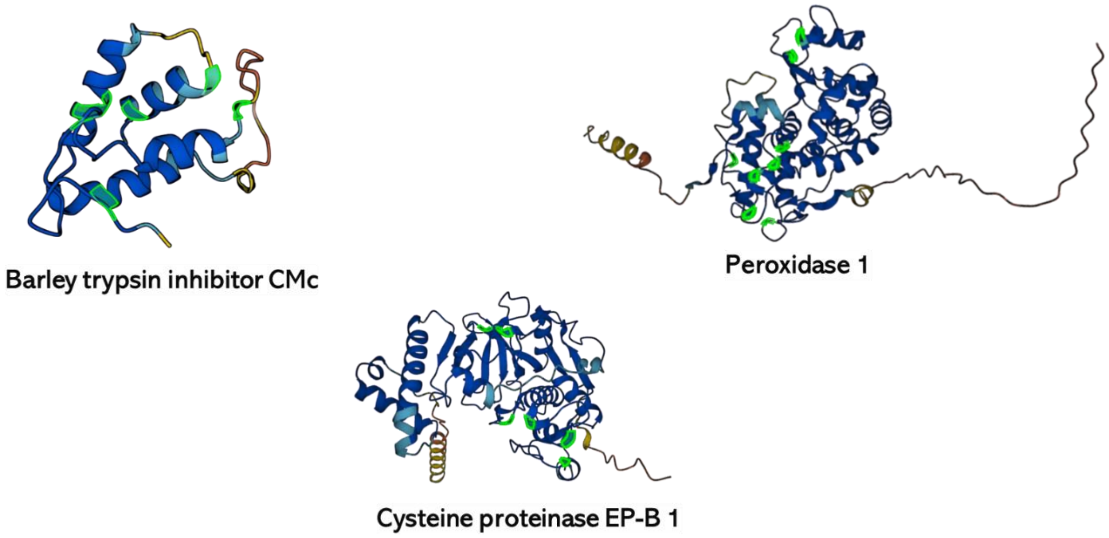


**Figure S6.** 3D Structure of native isoforms of barley protein. Dissulfide bonds are highlighted in green.
*Structure extracted from the AlphaFold database*[1,2].

## REFERENCES


(1) Jumper, J.; Evans, R.; Pritzel, A.; Green, T.; Figurnov, M.; Ronneberger, O.; Tunyasuvunakool, K.; Bates, R.; Žídek, A.; Potapenko, A.; Bridgland, A.; Meyer, C.; Kohl, S. A. A.; Ballard, A. J.; Cowie, A.; Romera-Paredes, B.; Nikolov, S.; Jain, R.; Adler, J.; Back, T.; Petersen, S.; Reiman, D.; Clancy, E.; Zielinski, M.; Steinegger, M.; Pacholska, M.; Berghammer, T.; Bodenstein, S.; Silver, D.; Vinyals, O.; Senior, A. W.; Kavukcuoglu, K.; Kohli, P.; Hassabis, D. Highly Accurate Protein Structure Prediction with AlphaFold. *Nature* **2021**, *596* (7873), 583–589. https://doi.org/10.1038/s41586-021-03819-2.

(2) Varadi, M.; Anyango, S.; Deshpande, M.; Nair, S.; Natassia, C.; Yordanova, G.; Yuan, D.; Stroe, O.; Wood, G.; Laydon, A.; Zídek, A.; Green, T.; Tunyasuvunakool, K.; Petersen, S.; Jumper, J.; Clancy, E.; Green, R.; Vora, A.; Lutfi, M.; Figurnov, M.; Cowie, A.; Hobbs, N.; Kohli, P.; Kleywegt, G.; Birney, E.; Hassabis, D.; Velankar, S. AlphaFold Protein Structure Database: Massively Expanding the Structural Coverage of Protein-Sequence Space with High-Accuracy Models. *Nucleic Acids Res* **2022**, *50* (D1), D439–D444. https://doi.org/10.1093/nar/gkab1061.
